# The psychological burden of long-term care facilities personnel during the SARS-COV-2 pandemic – a national survey in poland

**DOI:** 10.1192/j.eurpsy.2021.709

**Published:** 2021-08-13

**Authors:** A. Senczyszyn, K. Lion, D. Szcześniak, E. Trypka, J. Mazurek, M. Ciułkowicz, K. Fila-Witecka, M. Pawłowski, M. Łuc, M. Maćkowiak, J. Rymaszewska

**Affiliations:** 1 Department Of Psychiatry, Wroclaw Medical University, Wroclaw, Poland, Wrocław, Poland; 2 Menzies Health Institute Queensland, Griffith University, Nathan, Australia; 3 Department And Division Of Medical Rehabilitation, Wroclaw Medical University, Wrocław, Poland

**Keywords:** COVID-19, long term care facilities, Psychological Distress

## Abstract

**Introduction:**

The high COVID-19 morbidity and mortality are observed among residents in long-term care facilities (LTCF) worldwide. Employees of LTCF, who are facing a critical epidemiological situation endangering the vulnerable residents, are exposed to pandemic’s psychological consequences daily.

**Objectives:**

The main aim of this study was to assess psychological consequences (somatic symptoms, anxiety and insomnia, social dysfunction, and depression) among LTCF employees exposed to the SARS-CoV-2 pandemic crisis. Moreover, we investigated if factors such as: personal protective equipment (PPE) availability, safety guidelines or access to psychiatric and psychological support at the workplace, correlated with the level of psychological distress experienced by personnel.

**Methods:**

A cross-sectional study was conducted among personnel of LTCF in Poland. The survey consisted of the sociodemographic section, the authors’ questionnaire with questions related to COVID-19 exposure, working conditions, access to PPE and mental health services, GHQ Questionnaire-28.

**Results:**

show that access to PPE (P= .018), to workplace safety guidelines (P= .031), psychological support at workplace (P<0.01), fixed shift schedule (P= .05) and feeling that the right number of staff are employed in the workplace (P= .009), were related to the lower severity of psychopathological symptoms evaluated with the GHQ-28.

**Conclusions:**

The study indicates an evidence that LTCF personnel are susceptible to the development of anxiety, depression, insomnia and social dysfunction during the pandemic crisis. However, these can be modified by: access to PPE, safety guidelines and psychological support. Findings from this study lay a basis for effective interventions aiming to support psychological health within this group.

